# Microbiome and pregnancy: focus on microbial dysbiosis coupled with maternal obesity

**DOI:** 10.1038/s41366-023-01438-7

**Published:** 2023-12-25

**Authors:** Kalie F. Beckers, Juliet P. Flanagan, Jenny L. Sones

**Affiliations:** 1 Veterinary Clinical Sciences, School of Veterinary Medicine, Louisiana State University, Baton Rouge, LA USA; 2 Pennington Biomedical Research Center, Louisiana State University, Baton Rouge, LA USA; 3 Present Address: Clinical Sciences, Colorado State University College of Veterinary Medicine and Biomedical Sciences, Fort Collins, CO USA

**Keywords:** Obesity, Obesity

## Abstract

Obesity is becoming a worldwide pandemic with over one billion people affected. Of women in the United States, who are of childbearing age, two-thirds of them are considered overweight/obese. Offspring of women with obesity have a greater likelihood of developing cardiometabolic disease later in life, therefore making obesity a transgenerational issue. Emerging topics such as maternal microbial dysbiosis with altered levels of bacterial phyla and maternal obesity programming offspring cardiometabolic disease are a novel area of research discussed in this review. In the authors’ opinion, beneficial therapeutics will be developed from knowledge of bacterial-host interactions at the most specific level possible. Although there is an abundance of obesity-related microbiome research, it is not concise, readily available, nor easy to interpret at this time. This review details the current knowledge regarding the relationship between obesity and the gut microbiome, with an emphasis on maternal obesity.

## Introduction

According to the National Institutes of Health and the Worldwide Health Organization (WHO), obesity is becoming a global pandemic. The obesity rate in the United States (US) alone was 41.9% in 2020, with an estimated 1 billion people classified as obese worldwide. Body mass index (BMI) is the current internationally accepted measurement of obesity. It is calculated as body weight (in kg) divided by body height (in m^2^). People classified as overweight, obese, and morbidly obese are those with a BMI of 25–30 kg/m², 30–40 kg/m², and greater than 40 kg/m², respectively. People classified as underweight and of normal weight are those with BMI of less than 20 kg/m² and 20–25 kg/m², respectively. Obesity increases the risk of cardiovascular and other diseases, compromises the quality of life, and increases overall mortality [1].

Obesity is a complex metabolic disorder that is associated with insulin resistance, hyperglycemia, hyperlipidemia, and hypertension, and is closely linked to the most common human conditions including diabetes, cardiovascular diseases, and cancer [2]. Simply put, obesity can be interpreted as an abnormal deviation from the normal energy steady state [3]. Adipose tissue actively participates in systemic inflammation and immunity by producing and releasing a variety of pro-inflammatory and anti-inflammatory factors [4]. In the obesogenic environment, an excess of white adipose tissue releases increased amounts of inflammatory cytokines, creating a systemic pro-inflammatory state [5]. It is well known that obesity is characterized by an increased level of some adipokines such as leptin, resistin, visfatin, and a reduction of adiponectin [6]. Gut microbiota may affect host metabolism via microbiome metabolites [7]. These microbial metabolites are key for energy acquisition and can metabolize dietary nutrients into many bioactive substances, thus acting as a link between the gut microbiome and its host. They include but are not limited to short chain fatty acids (SCFAs), bile acids, ethanol, and amino acids [8]. For example, reduced levels of *Bifidobacterium* correlates with decreased production of butyrate and folate bioavailability [9]. Additionally, *Lactobacillus* reduction also correlates with decreased production of butyrate [9].

Of the women in the US who are of childbearing age, two-thirds of them are considered overweight/obese, and nearly half of these women have excessive gestational weight gain once pregnant [10]. Maternal obesity is linked to unfavorable pregnancy outcomes such as gestational diabetes, gestational hypertension, preeclampsia, and caesarean section [11]. Excess maternal weight gain is a predisposing factor for the development of high birth weight and obesity in the offspring [12], in addition to other severe consequences such as preterm birth, congenital defects, perinatal death, and metabolic diseases [13]. The ongoing hypothesis is that improving maternal metabolism through maternal diets and altering the gut microbiome composition may benefit the transgenerational burden of disease. Hence, evaluation of the gut microbiome may lead to beneficial outcomes such as prevention of obesity in the mother and potentially in the offspring through fetal programming. Thus, the purpose of this meta-analysis is to clarify the current knowledge regarding maternal gut microbiome, obesity, microbial dysbiosis and their transgenerational effects.

## Methods

Each author individually retrieved articles up to July 1, 2023, by searching PubMed using the following search terms: ‘obesity’, ‘gut microbiome’, ‘maternal obesity’, ‘maternal microbiome’, ‘gut microbiome inflammation,’ and ‘effect of obesity on offspring’. The reference lists of relevant articles and reviews were also searched manually. The search of obesity and the gut microbiome yielded over 5000 articles. This was reduced to 2289 by only comparing articles from 2000 to 2023, removing anything except original research papers and relevant review articles. Then, any research article coupled with confounding disease was removed. Articles using next generation sequencing for microbiome analysis were selected. Similar search methods were performed on ‘maternal obesity’ and ‘gut microbiome’ yielding 288 results. Additionally, ‘effects of obesity on offspring’ was searched, yielding 856 results before removing confounding disease studies.

## Function of gut microbiome and how its impaired with obesity

A microbiome is a collective genomic community of symbiotic, commensal, and sometimes pathogenic microorganisms that reside in an environment, such as a body cavity. These communities can consist of bacteria, archaea, fungi, protists, and viruses. The host and microbiome relationship is considered a mutualistic symbiosis [14]. Maruvada et al., coined the gut microbiome as a ‘microbial organ’ that responds to environmental, dietary, and host factors, that ultimately plays a role in host physiology and pathophysiology [3]. Differences in composition and function of the gut microbiome have been associated with obesity in humans, as well as other species [15].

The functions of the gut microbiome are essential for life. The commensal gut microbiota protects against pathogenic bacteria, metabolizes indigestible polysaccharides, and produces vital nutrients such as short chain fatty acids (SCFA) [16]. SCFAs are the main fermentation products of the digestion of insoluble fibers. The primary SCFAs produced in the gut are acetate, propionate, and butyrate. The gut microbiota plays a critical role in fat production. Bacterial SCFAs are used by the host for lipid synthesis, therefore providing additional calories [17]. A study by Bäckhed et al. found that, through de novo lipogenesis, colonizing male and female, germ-free, C57, juvenile mice from microbiota from conventional mice dramatically increased body fat, despite a decrease in food consumption [18]. An even more dramatic example is the colonization of germ-free mice from mice with genetic obesity, which caused a greater increase in body fat than transfaunation from lean mice [19]. Varying bacterial populations produce different substrates and metabolites, resulting in differing capacities for energy harvest. Namely, Firmicutes and Bacteroidetes have been investigated the most thoroughly [19, 20].

Throughout the literature, Firmicutes have been coupled with obese microbiome, while Bacteroidetes have been denoted to be considered healthier [20–22]. Mice with genetic obesity (*ob/ob*) demonstrate shifts in the ratio of Firmicutes to Bacteroidetes abundances, with Bacteroidetes significantly reduced, and Firmicutes increased in the murine obesity model [23]. These results are consistent with the findings of Turnbaugh et al., which showed that the gut microbiome of mice with obesity expressed more genes that encode for enzymes involved in energy extraction, allowing mice with obesity to harvest more energy from their diet compared to lean controls [19]. Furthermore, excess energy intake favors the proliferation of Firmicutes over Bacteroidetes in humans [24]. Several studies report differing overall abundances of the Bacteroidetes and Firmicutes phyla in the intestinal microbiota of participants characterized as obese and lean. Multiple sources report a decreased relative abundance of bacteria belonging to Bacteroidetes phylum, as evidenced by depletions of the family *Rikenellaceae* [25] and the genus *Barnsiella* [26] in subjects with obesity (Table 1). In conclusion, controversial literature states that this may not always be the case, instead we need to focus on potentially their ratio or more specific taxa within those phyla [17, 27]. More importantly to look at specific taxa at a lower classification to distinguish a microbial dysbiosis, which can lead to changes further down in the host metabolic processes. For example, Schwiertz et al. found that human subjects with obesity had a 20% higher total amount of SCFAs with approximately 2–3% higher proportion of propionate than lean groups [17].

**Table 1:**
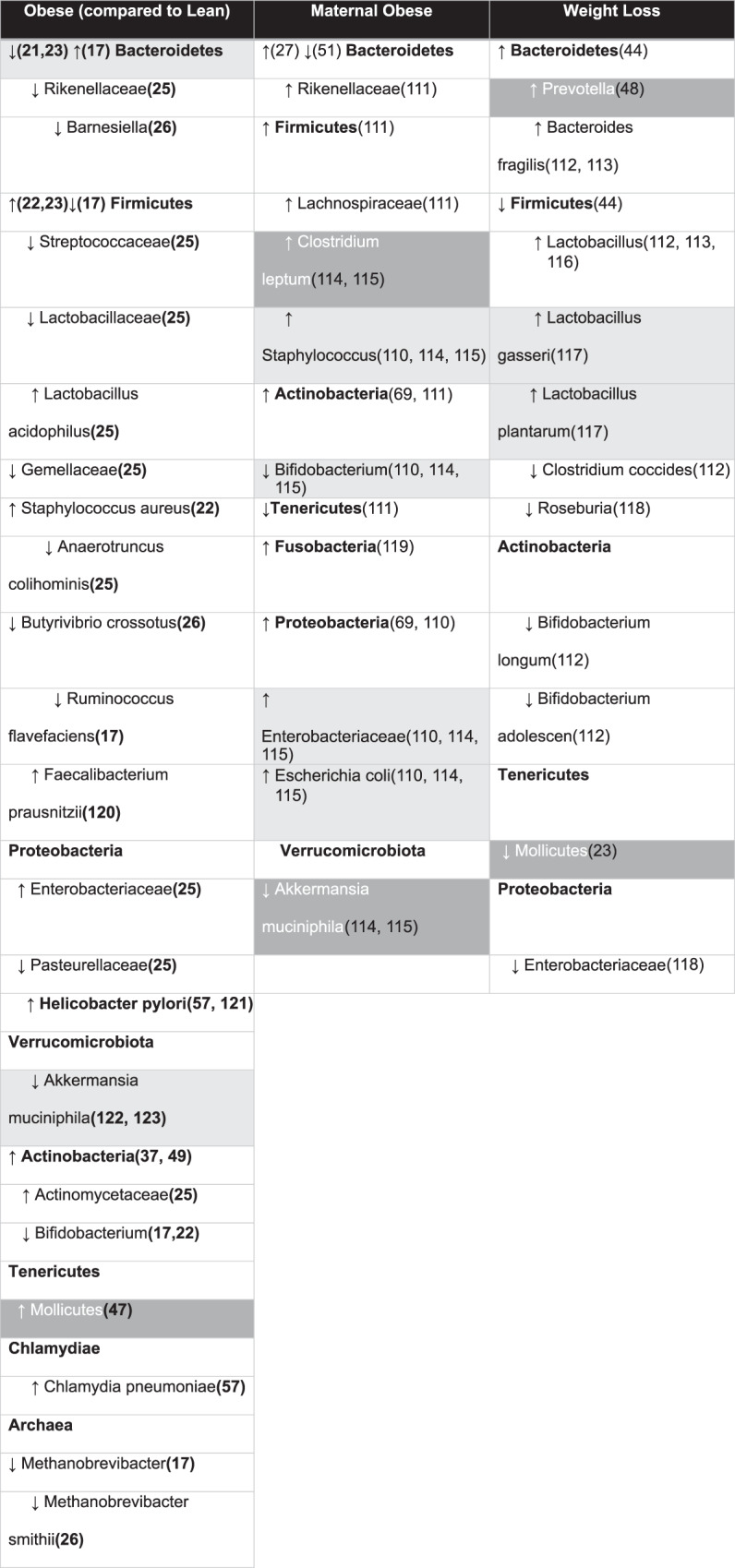
Specific microbiome changes associated with obesity [17, 22, 23, 25, 26, 37, 44, 47–49, 57, 69, 47–49, 110–123].

Microbes from human studies are written as plain text, black letters with white background. Microbes from animal studies are highlighted in gray with white letters. Microbes found in both human and animal studies are highlighted in dark gray with black writing. Phyla is bolded, **↑** denotes increased and ↓ denotes decreased.

SCFAs are one of the major metabolites produced by gut microbiota during food fermentation, and levels of SCFAs depend on the composition of the gut microbiota [28]. Finally, the gut microbiome yields energy for the host’s metabolism from the production of SCFAs [29]. Puertollano et al. suggested that SCFAs, specifically increased propionate and butyrate, may contribute to inflammatory and metabolic disorders, including obesity [28]. SCFAs may have a beneficial effect on host metabolism and appetite control [30]. When considering external influences on the gut microbiome, diet is one of the most important factors that affect microbial community, diversity, and structure, which impact a variety of host metabolic responses [3]. For example, high-fat diets have consistently altered composition and richness of the gut microbiota [31].

## Microbial dysbiosis and its effects

The gut microbiota is also considered a regulator of energy homeostasis. A disruption of the host’s intestinal microbiota, known as “gut dysbiosis,” is closely linked to the development of inflammatory disease such as obesity [32, 33] According to the literature, exposure of the intestinal microbiota to certain environmental insults can result in energy homeostasis impairment and the eventual development of obesity [34].

Several studies in animal models have demonstrated the impact of diet on gut microbial communities. It has been hypothesized that the changing microbial community composition alter critical host-microbe interactions on a real-time basis [35]. Weight loss intervention studies have shown an association with shifts in microbiota composition, reduction in weight, and improvement in metabolism [3]. In another intervention, fecal microbiota transfers (FMT) into recipient human and rodent hosts have resulted in mimicked phenotypes of the donor [36]. A core set of microbes were found to be associated with lean body composition; likewise, microbial communities associated with weight gain (See Table 1) and metabolic disease were also characterized [37, 38]. Still more research is needed to completely understand the connection between the gut microbiota and obesity development.

## Key players/changes in microbiome

Sequela of microbial dysbiosis of the gut microbiome includes diseases such as allergies [39], inflammatory bowel disease [40], diabetes type 1 and 2 [41, 42], and metabolic syndrome [20]. These studies indicate a causative role the gut microbiome may play in disease pathogenesis. Firmicutes and Bacteroidetes consistently make up the majority of bacteria found within the gut of both humans and mice. Together, they typically comprise over 90% of the gut microbiome [43]. An inappropriate ratio and composition of these phyla in the gut is found in animals with obesity across various species. For example, mice with decreased Bacteroidetes and increased Firmicutes were more likely to develop obesity [23]. Decreased levels of Bacteroidetes were also found in swine with obesity [21] (Table 1). In a comparable human study, children who became overweight by the age of 7 years had an increased amount of Firmicutes compared to lean children [22].

Several investigations found that these changes in Firmicutes and Bacteroidetes composition are reversed with weight loss (Table 1). A human adolescent study found decreased Firmicutes and increased Bacteroidetes during weight loss of individuals with obesity [44]. Additionally, decreased Firmicutes populations have been associated with weight loss in patients following gastric bypass surgery [45]. Turnbaugh et al. found that 75% of obesity related genes were from Actinobacteria and 25% were from Firmicutes, while 42% of lean-enriched gene were found Bacteroidetes [37]. Turnbaugh et al. also showed that a switch from a low-fat diet to a high-fat, high-sugar diet in mice, which was associated with obesity, increasing Firmicutes and decreasing Bacteroidetes in the gut within one day [38] (Table 1).

The Firmicutes phylum comprises gram-positive organisms from greater than 200 different genera including *Catenibacterium, Clostridium, Eubacterium, Dorea, Faecalibacterium, Lactobacillus, Roseburia, Ruminococcus*, and *Veillonella* while the Bacteroidetes phylum consists of gram-negative bacteria from approximately 20 genera including *Bacteroides, Odoribacter, Prevotella, and Tannerella* [46] (Table 1). Studies in animals have shown similar results: introducing a western high-fat, high-sugar diet to mice led to increased *Mollicutes*, a Firmicutes, and caused a suppression of Bacteroidetes [47]. Microbial transplantation of this *Mollicute*-rich flora into germ-free mice led to higher adiposity than transplantation of flora from lean mice, suggesting that the restructured flora may promote superior processing of sugars and fat [47]. Weight loss in humans with obesity has been associated with a decrease in *Mollicute* predominance and with an associated increase in the abundance of Bacteroidetes [23] (Table 1). In another example, the *Prevotella* genus has been found to be inversely correlated with body weight gain, cholesterol accumulation, insulin resistance and diet-induced adiposity [48] (Table 1). Microbes from the *Lachnospiraceae* family have been found to be dominant in fecal samples from both lean mice and humans. In mice, the most abundant genera found in a healthy core microbiome are an unclassified genus from the *Porphyromonadaceae* and *Alistipes*. However, in humans core microbiome, *Roseburia* and an unclassified genus representing the *Clostridiales* order are the most abundant [43] (Table 1).

Peters et al. found that at the phyla level there was no significant difference in the relative abundances of Firmicutes and Bacteroidetes between BMI classes. However, differential abundance of several classes and families within the Firmicutes phylum were associated with obesity. The *Bacilli* class, along with the families *Streptococcaceae*, *Lactobacillaceae*, and *Gemellaceae* had higher abundances in participants with obesity compared to healthy-weight participants [25]. There is also differential abundance within the class *Clostridia*, a sub-taxa of the Firmicutes phyla; the *Veillonellaceae* family was enriched in participants with obesity while the *Christensenellaceae*, *Clostridiaceae*, and *Dehalobacteriaceae* families were depleted [25]. Within the phylum of Proteobacteria, increased abundances of family *Enterobacteriaceae* were found in participants with obesity, along with decreased abundances of family *Pasteurellaceae* [25]. Overall, the phylum Actinobacteria was elevated in the human gut microbiome of humans with obesity, with the family *Actinomycetaceae* being representative of this increased abundance [25, 37, 49]. However, a decreased relative abundance of the genus *Bifidobacterium* [17, 22] within the Actinobacteria phylum was found in fecal samples from adults and children with obesity. Archaea is also found within the human gut microbiome. A decreased relative abundance of the genus *Methanobrevibacter*, specifically *Methanobrevibacter smithii*, is associated with obesity [17, 26] (Table 1).

## Linking obesity to inflammation

Obesity has been shown to increase levels of pro-inflammatory cytokines by increasing the number of macrophages and their infiltration in adipose tissue. Nieta et al., characterized obesity as an inflammatory state by which there is a positive correlation between fat mass and the expression of tumor necrosis factor alpha (TNFa) as well as other pro-inflammatory cytokines [50]. Lipopolysaccharides (LPS), a metabolic endotoxin commonly found in the intestines, has been known to cause systemic inflammation. Cani et al. found that a high-fat diet increased the proportion of gram-negative bacteria in the gut and more than doubled plasma LPS concentrations, thereby inducing metabolic endotoxemia in mice within four weeks of the diet change. Furthermore, the fat-enriched diet led to increased expression of pro-inflammatory cytokines TNFa, interleukin-6 (IL-6), and interleukin-1 (IL-1) in visceral and subcutaneous fat depots [51]. Healthy gut microbiota may mitigate the risk of obesity caused by LPS [52]. These human and animal studies have shown that gut microbes may influence adiposity and weight gain by affecting host gene expression and impacting metabolic and inflammatory pathways [53, 54].

A potential explanation for exacerbation of microbial dysbiosis and increased inflammation coupled with obesity is a decreased host immune function. For example, decreased activity in macrophages, dendritic cells, and natural killer cells is found in individuals with obesity [55]. In Zucker rats, the rats with obesity retained and carried a higher burden of *Candida* compared to the lean controls due to decreased bactericidal activity of all phagocyte populations [56]. In humans, *Helicobacter pylori* and *Chlamydia pneumoniae* are increased in individuals with higher BMI and higher fasting insulin concentrations [57]. Especially in women, *Chlamydia pneumoniae* IgG antibodies were related to waist and hip circumference [58].

Wang et al. [59]. showed that specific gut microbial components from gram-negative microbes could interact with innate immune cells, initiating cytokine release, which leads to energy balance disruption in the host. Certain bacterial taxa have been shown to be anti-inflammatory and protective against host visceral fat deposition (*Akkermansia muciniphila* [60] for example), whereas absence of such bacterial taxa has been implicated and demonstrated in obesity [3].

## Maternal obesity and the effects on pregnancy

Obesity, pre-conception and throughout pregnancy, affects more than just the mother; the effects can be lifelong for the offspring as well. Even though mechanisms are poorly understood, maternal obesity is a key predictor of childhood obesity and may predispose weight gain in the offspring [61]. One of the functions of the gut microbiota is to modulate fat deposition. Due to their metabolic and immunological regulatory roles, including regulation of energy extraction [19], immunity [62], and lipid metabolism [63], a dysbiosis in the gut microbiota can impair host metabolism and result in obesity [63]. It seems like a plausible hypothesis that the maternal gut microbiome would have downstream effects on pregnancy outcomes by (i) maternal adaptation to pregnancy (ii) altered placental function, and (iii) changes to fetal intrauterine environment due to impaired fat deposition [61].

**Fig. 1:**
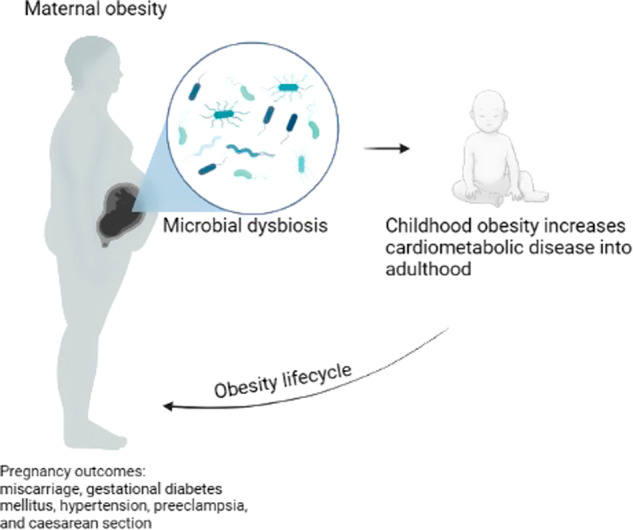
Transgenerational effects of maternal obesity on pregnancy and childhood outcomes. The microbial dysbiosis associated with obesity may influence the gut microbiome of the fetus *in utero* and predispose the offspring to cardiometabolic disease later in life. *Created with Biorender*.

During a healthy pregnancy, the maternal body undergoes dramatic physiological changes to support the growth of fetus and the placenta. Maternal gut dysbiosis may disturb the remodeling of physiological balance, leading to maternal maladaptation. Pregnant women with this potential maladaptation are at high risk of developing pregnancy disorders, which is increasingly observed in clinical cases [64]. Adding obesity to pregnancy increases the risk for pregnancy complications, such as miscarriage, gestational diabetes mellitus (GDM), hypertension, preeclampsia, and caesarean section [65] (Fig. 1). Both preeclampsia and gestational hypertension have been reported to increase linearly with maternal BMI. Therefore women with obesity have a 3-fold higher risk of developing these complications [66]. Maternal obesity is also associated with significant changes in endocrine and metabolic function where circulating levels of SCFAs are dysregulated [67] along with increased pro-inflammatory cytokines [68]. Previous studies demonstrate that women who were overweight or obese prior to pregnancy and subsequently develop GDM also had a significant shift in beta-diversity in their gut microbiome from their 1st trimester to their 3rd [69]. Although the composition and diversity of the gut microbiome during the first trimester is similar to that of non-pregnant healthy women, over the course of pregnancy there is an increase in the abundance of Proteobacteria and Actinobacteria [69]. The intestinal microbiota composition during pregnancy has been shown to be affected by pre-pregnancy weight and weight gain over the course of gestation, which will display individual variation [61]. An accepted hypothesis about the effect of maternal obesity on offspring involved the role of the maternal gut bacteria composition, which is critically dependent on the host’s nutrition [61]. Since the bacteria are responsible for modulating weight gain, metabolism, regulation of energy extraction, and lipid metabolism, microbial dysbiosis would be detrimental to the developing fetus. Exploration of basic mechanisms of this process in pregnancy need to be performed.

## Maternal microbiome and the placenta

In contrast to the once thought “sterile placenta” concept, there is emerging research demonstrating that a microbiota exists in the placenta, amniotic fluid, umbilical cord blood, and meconium in healthy pregnancies [70, 71]. The in-utero colonization hypothesis suggests that during gestation, the low bio-mass placental microbiome colonizes the fetus as it develops [72]. This indicates that the offspring’s microbiota may be acquired from the mother’s microbiome that inhabits the *in utero* environment prior to birth [73, 74]. Yang et al., demonstrated that the microbiome of offspring is determined by genetic and intrauterine factors [75]. Based on this study by Li et al., there is evidence to support a hypothesis of vertical transmission of a dysbiotic microbiome across the placenta to colonize the fetus prior to birth. This is a proposed pathway for the contribution of microbial dysbiosis in the cycle of transgenerational obesity [76]. However, the precise route of transmission remains unclear and needs further research.

There are several proposed mechanisms for in-utero colonization of offspring. Aagaard et al. suggested that the maternal oral cavity may contribute to systemic bacterial counts that localize within the placenta possibly during implantation through hematogenous transfer [70]. Further evidence that the oral cavity and placenta microbiotas interact includes elevated inflammatory responses and exacerbated bacterial disease, such as gingivitis, during pregnancy [77, 78].

A common sequela of maternal obesity is gut dysbiosis and “leaky gut syndrome”, which is defined as the weakening or failure of the epithelial barrier of the gut caused by stress, chronic inflammation, and dysbiosis of the gut microflora [14]. Leaky gut syndrome can increase bacterial translocation during pregnancy. An example of this is bacteria from the gut of pregnant mice were found in mesenteric lymph nodes and are thought to be transferred through the placenta into the fetus, where bacteria have been demonstrated in meconium [71].

A potential mechanism of action, maternal BMI may be associated with activation of placental inflammatory pathways via immune cells and pro-inflammatory cytokines [61]. This suggests that an elevation in maternal BMI with obesity leads to an increase in pro-inflammatory cytokines in maternal plasma and activation of placental inflammatory pathways. This may influence placental function leading to hypoxemia and restricted perfusion resulting in adverse outcomes. It has also been found that placentas from obese women have developed an exaggerated inflammatory response that includes macrophages infiltration via monocyte chemoattractant protein (MCP)−1 and tumor necrosis factor-alpha [61]. It has been hypothesized that this modulates placental nutrient transfer negatively. An example of this is placentas of obese women demonstrate decreased sodium-dependent neutral amino acid transporter expression [61]. Additionally, in vitro, IL-1β has been found to be inhibited by insulin-stimulated System A amino acid uptake in human placental trophoblast cells [61]. Obese women have also shown placental dysregulation of redox balance, decreased fatty acid transport, and increased placental lipoprotein lipase activity which could be responsible for placental triglyceride accumulation [61]. Demonstrating potential mechanisms by which maternal obesity affects the placenta, which has in-utero downstream consequences on the offspring.

Looking further into the mechanism of maternal obesity effects on the offspring develop, a functional consequence of a dysbiotic gut would be changes in SCFA production. A study by Chen et al., demonstrated that SCFAs are related to the progression of GDM, PE, and intrahepatic cholestasis of pregnancy (ICP) [79]. An analysis comparing non-pregnant women to pregnant women showed that the concentration of isobutyric acid in the pregnancy complications groups was significantly higher, and the levels of other SCFAs were also significantly different from those in the non-pregnant group. Acetic, propionic, and isobutyric acids were found to be higher with the incidence of PE [80]. In addition, SCFAs were closely related to the increased risk of ICP, more specifically caproic acid and isobutyric acid. Further analysis showed that SCFAs significantly correlated with the general characteristics of the mothers and various clinical indicators; therefore, supporting SCFAs as an emerging avenue to study potentially as new biomarkers for clinical diagnosis and monitoring.

## Maternal microbiome transgenerational effects on offspring

Maternal obesity is currently one of the leading factors predicting childhood obesity [81] (Fig. 1). Maternal obesity is associated with abnormal feto-placental function [82], offspring obesity risk [81], and increased disease risk in general [61]. Catalano et al. found fetuses of mothers with obesity had greater percent body fat and developed insulin resistance *in utero* [83]. Other developmental abnormalities such as neural tube defects are more common in infants born to women with obesity [84].

After birth, the distinct microbiome signature of the meconium of newborn infants is altered by maternal metabolic and health status [85]. The presence of specific strains of maternal enteric bacteria in the meconium implies that the fetus is exposed to microbes from the maternal gut *in utero* [86] (Fig. 1). Although excess maternal weight gain causes dysbiosis of the gut microbiota, little is known about the impact of this dysbiosis on the maternal or fetal metabolism during pregnancy [61]. Studies have shown that obesity can be inherited and the development of obesity can be influenced *in utero* [87, 88] (Fig. 1).

Further evidence of maternal to fetal transmission of bacterial microbes comes from studies investigating the transmission of specific maternal bacteria to the fetal gut. Makino et al. showed that specific *Bifidobacterium* strains found in the maternal intestines colonize the infant’s gut for up to 90 days post birth [89]. Ma et al. stated that early infant gut microbiota can be influenced by maternal diet during pregnancy and lactation, concluding that early exposure to a high-fat diet disrupts the infant’s commensal microbial communities, which are not completely rebalanced by being fed a low-fat diet afterwards [90]. Koren et al. found that regardless of age, the microbiome of offspring are most similar to their mother’s microbiota from the first trimester [69].

The development of obesity does not just affect the offspring during infancy, it has lifelong effects (Table 1). Increased metabolic activity of the gut microbiota has been found in children with obesity compared to lean and is involved in the etiology of the lifelong obesity [91]. Increased metabolic activity is measured by the number of active enteric bacteria and the amount of SCFAs produced [91]. Shifts in the microbial populations of children with obesity exist even at the species level [92]. For example, children with obesity have a three times greater risk of being carriers of *Neisseria* than non-subjects with obesity, and this risk increases with severity of BMI [93]. These studies demonstrate how the maternal microbiome permanently influences the offspring, with an obesogenic and dysbiotic maternal microbiome having deleterious effects on the health of the offspring (Fig. 1).

## Probiotics and maternal obesity

Probiotics hold potential as a safe therapeutic tool for the prevention of pregnancy complications and adverse outcomes related to maternal obesity. A large Norwegian observational study “The Mother and Child Cohort Study”, observed the incidence of PE among pregnant women who reportedly consumed milk-based probiotics containing *Lactobacillus* bacteria. It was shown that the Lactobacillus probiotic may have suppressed the Gram-negative bacterial LPS expression to reduce inflammation [94]. In a clinical trial in non-pregnant individuals, involving the similar use of milk-based probiotics have reported reduced blood pressure effects [94]. Daily consumption of a probiotic containing *Lactobacillus* and *Bifidobacterium* species from 28 to 37 weeks gestation significantly lowered serum levels of high sensitivity C-reactive protein via enzymatic blocking of its hepatic synthesis, while consumption of the conventional yoghurt had no significant effect [94]. The probiotic containing *Lactobacillus rhamnosus* and *Bifidobacterium lactis* significantly reduced total HDL and LDL cholesterol, as well as triglyceride levels in obese pregnant women [94]. Another study reported that blood glucose concentrations during pregnancy were lowest in the diet/probiotic group and this group also had better glucose tolerance, as evidenced by a reduced risk of elevated glucose concentration compared to the control group [94]. In conclusion, the studies demonstrate several beneficial effects of probiotic consumption in pregnancy, indicating their potential use as a safe therapeutic tool to improve maternal outcomes, especially obesity associated adverse pregnancy outcomes.

## Obesity and Inflammation during pregnancy

**Fig. 2:**
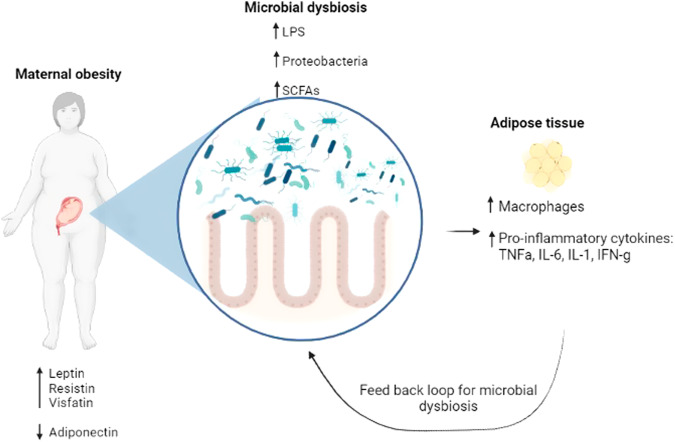
Hypothesis correlating maternal obesity, microbial dysbiosis, and inflammation. Inflammation due to pregnancy affects microbial metabolism and composition, resulting in a dysbiosis and increased fat deposition. These effects are exacerbated by the systemic inflammation and microbial dysbiosis associated with obesity. *Created with Biorender*.

The original theory was that an overgrowth or dysbiosis of Bacteroidetes and/or Firmicutes led to the development of maternal obesity which also may lead to an increase in inflammatory cytokines contribute to adverse pregnancy outcomes, such as preeclampsia [14]. Lupp et al. suggests that low grade inflammation develops during pregnancy and drives the microbial dysbiosis into a positive feedback loop with an altered host response [95] (Fig. 2). A hypothesis of the contribution of the gut microbiota to host adiposity during pregnancy is that there are altered host microbial interactions that promote metabolic inflammation [69], with dysbiosis driving changes to the gut microbiome metabolism that ultimately affects fat deposition in the host (Fig. 2). For example, there was a higher average proportion of Proteobacteria, specifically *Enterobacteriaceae*, during the 3rd trimester of pregnancy. Increased Proteobacteria have been associated with a greater inflammatory response. In a study by Koren et al., levels of proinflammatory cytokines IFN-g, and TNF-a were significantly higher in the 3rd trimester, matching the increased levels of Proteobacteria [69]. During pregnancy, elevated levels of circulating cytokines (TNF-a and IL-6) [96] are thought to drive obesity-associated metabolic inflammation [97] (Fig. 2). In a healthy pregnancy, an increase in the abundance of Proteobacteria is found from the 1st trimester to the 3rd, which has been observed repeatedly for inflammation-associated dysbiosis [98]. Since an increase in Proteobacteria has been observed in inflammatory bowel disease, it has been suggested that a similar dysbiosis of the gut occurs during the third trimester of pregnancy [98]. Pro-inflammatory cytokines (such as IL-6 and TNF-a) are notably increased in stool samples collected from women during the third trimester in comparison to the first trimester [69]. These maternal microbiome modifications could lead to an aberrant intrauterine environment that promotes altered gut development and increased chronic disease risk in offspring (Fig. 2).

Leptin has been shown to activate neutrophils and T lymphocytes, increase cytokine productions, regulate activity of macrophages, and adversely effect on wound healing [99, 100]. Obesity can cause inhibitory signals to be blunted by leptin resistance, and can result in hyperphagia despite elevated circulating leptin levels [3]. If this hyperleptinemia and leptin resistance occurs during pregnancy, it can also have transgenerational effects on offspring. In a study by Catalano et al., fetuses born to mothers with obesity demonstrated leptin resistance and an increase in cord leptin and IL-6 [83].

*Ob/ob* mice, which are leptin deficient, have a severe obese phenotype with increased sensitivity to proinflammatory macrophages, impaired phagocytotic functions, and reduced T cell function [101]. These mice are highly susceptible to bacterial infections by *Listeria monocytogenes* and *Klebsiella pneumoniae* [102, 103]. New studies suggest that inflammation mediated by gut microbes may aggravate adipose tissue inflammation via increased gut permeability and enhanced circulating LPS levels, all potentially exacerbated by obesity [104]. This shows obesity may predispose for bacterial infection by microbial dysbiosis and demonstrate increased inflammation. One study found that administration of LPS to pre-gestation mouse dams resulted in hyperphagia, hyperleptinemia, and obesity in the female offspring [105]. Like obesity, LPS-induced inflammation before and during pregnancy may have lifelong effects on offspring.

Leukocytes and neutrophils have also been found to infiltrate the gut lumen [106]. One hypothesis regarding bacterial translocation from the intestinal tract to the placenta is thought to be through the blood stream by hematogenous transfer. Changes in maternal intestinal permeability are likely to play a role in bacterial translocation [61]. However, immune populations of dendritic cells may also promote maternal bacteria translocation [106]. During pregnancy and lactation, dendritic cells directly sample the luminal bacteria, which has been shown to contribute to the increased leakiness of the epithelium [107]. Dendritic cells have been found to have an increase in bacterial load during pregnancy and lactation [108] suggesting that they may be involved in bacterial translocation. When dysbiotic bacteria translocate to the placenta, it may cause increased inflammation. Maternal BMI has been associated with the activation of placental inflammatory pathways and increased pro-inflammatory markers MCP-1 and TNFa and has been shown to impair placental function [68]. Another study reported that placentas from women with obesity develop exaggerated inflammatory responses with increased macrophages infiltration and pro-inflammatory cytokines (TNFa and IL-6.) [109]. It is hypothesized that this pro-inflammatory environment may have the capacity to modulate placental nutrient transfer [61]. This could potentially create further dysbiosis leading to a vicious cycle of increased inflammation.

## Conclusions

In conclusion, obesity has been associated with microbial dysbiosis demonstrated by compositional changes and altered bacterial community diversity seen at the phyla level all the way down to the genus level. A decreased immune function has been associated with obesity which may be attributed to the development of microbial dysbiosis, in which the host does not create an appropriate immune response. As for the microbial dysbiosis within the gut, the main hypothesis links individuals with a microbial community as more efficient at energy extraction from the diet to lean individuals, and individuals with an increased ability to promote adiposity through manipulation of host genes and metabolism, may be predisposed to obesity. This hypothesis predicts that individuals with obesity will have distinct microbiotas from lean subjects, with measurable differences in their ability to extract energy from their diet and to deposit that energy as fat [37]. An example, germ-free mice are resistant to diet-induced obesity caused by consumption of a high-fat/high-sugar ‘Western’ diet showing how microbial dysbiosis is similar to an infectious cause of obesity. More research needs to be done to determine how to manipulate these bacterial communities back to the healthy state.

In summary, pregnancies cultivated with obesity are detrimental to the health of mother and baby. Women are encouraged to maintain a normal BMI and a balanced diet during pregnancy for their own health and the well-being of their offspring. It is possible that maternal gut dysbiosis as a result of obesity could ultimately affect the microbes the fetus is exposed to *in utero* and may serve to alter fetal gut development. It is this change in fetal gut microbiome composition that may potentially lead to changes in long-term gut function and metabolic compromise in the offspring. However, it is important to note that the presence of bacterial DNA is not the same as live bacteria; and thus far, live commensal bacterial populations have not been demonstrated to be present. Further research is needed to describe what specific microbial changes lead to maternal dysbiosis and altered pregnancy outcomes, and therapeutic approaches to actively correct these changes.
