# Correction: Microbiome and pregnancy: focus on microbial dysbiosis coupled with maternal obesity

**DOI:** 10.1038/s41366-025-01817-2

**Published:** 2025-06-16

**Authors:** Kalie F. Beckers, Juliet P. Flanagan, Jenny L. Sones

**Affiliations:** 1 Veterinary Clinical Sciences, School of Veterinary Medicine, Louisiana State University, Baton Rouge, LA USA; 2 Pennington Biomedical Research Center, Louisiana State University, Baton Rouge, LA USA; 3 Present Address: Clinical Sciences, Colorado State University College of Veterinary Medicine and Biomedical Sciences, Fort Collins, CO USA

**Keywords:** Obesity, Obesity

Correction to: *International Journal of Obesity* 10.1038/s41366-023-01438-7, published online 25 December 2023

In the section ‘KEY PLAYERS/CHANGES IN MICROBIOME’ of the original aritcle, the sentence “Turnbaugh et al. also showed that a switch from a low-fat diet to a high-fat, high-sugar diet in mice, which was associated with obesity, lowered the Firmicutes:Bacteroidetes ratio in the gut within one day [38] (Table 1)” now reads “Turnbaugh et al. also showed that a switch from a low-fat diet to a high-fat, high-sugar diet in mice, which was associated with obesity, increasing Firmicutes and decreasing Bacteroidetes in the gut within one day [38] (Table 1).” The original article has been corrected.

